# Impact of Rehabilitation Intervention for Cancer Patients with Spinal Bone Metastasis: Psychosocial and Clinical Outcomes

**DOI:** 10.3390/geriatrics10020056

**Published:** 2025-04-07

**Authors:** Noémi Németh, Lavinia Davidescu, Liviu Lazăr, Florica Voiță-Mekeres, Mariana Racoviță, Călin Tudor Hozan

**Affiliations:** 1 Doctoral School of Biomedical Sciences, Faculty of Medicine and Pharmacy, University of Oradea, 1 Universitatii Street, 410087 Oradea, Romania; nemeth.noemi@didactic.uoradea.ro (N.N.); lazarlv@yahoo.com (L.L.); voita-mekeres.f@uoradea.ro (F.V.-M.); 2 Department of Psycho-Neuroscience and Rehabilitation, University of Oradea, 410073 Oradea, Romania; 3 Department of Medical Disciplines, Faculty of Medicine and Pharmacy, University of Oradea, 1 Universitatii Street, 410087 Oradea, Romania; 4 Department of Morphological Disciplines, Faculty of Medicine and Pharmacy, University of Oradea, 1 Universitatii Street, 410087 Oradea, Romania; 5 Department of Surgical Disciplines, Faculty of Medicine and Pharmacy, University of Oradea, 1 Universitatii Street, 410087 Oradea, Romania; racovita.mariana@student.uoradea.ro (M.R.); chozan@uoradea.ro (C.T.H.)

**Keywords:** cancer, rehabilitation, psychosocial outcomes, vertebral metastasis, functional independence, coping mechanisms

## Abstract

**Background/Objectives:** Cancer remains a significant global health issue in the 21st century, accounting for 16.8% of all deaths and 22.8% of noncommunicable disease (NCD) deaths globally. This study investigated the impact of a novel integrated rehabilitation intervention on clinical and psychosocial outcomes in cancer patients with vertebral metastasis. **Methods:** The three-year study included newly diagnosed oncological patients or those undergoing treatment, aged 18 years or older, with vertebral metastasis and spinal pain. The intervention was tailored to each patient based on mental and functional reserves, risk of vertebral fractures, physical reserves, fatigue, and ongoing oncological therapy. **Results:** The control and experimental groups were compared in terms of baseline characteristics, physical activity, tumor characteristics, pain, sphincter disorders, complications, survival, functional scores, and coping mechanisms. The experimental group demonstrated significantly better outcomes, including longer mean survival time (3.5 vs. 2.8 years, *p* < 0.001), higher Barthel Total Score (60.7 vs. 40.8, *p* = 0.002), and lower prevalence of fractures (20.0% vs. 55.4%, *p* < 0.001), osteoporosis (17.0% vs. 37.0%, *p* = 0.001), anemia (22.2% vs. 57.4%, *p* < 0.001), and vomiting (6.1% vs. 54.5%, *p* < 0.001). The experimental group also exhibited a lower reliance on avoidant coping strategies (29.0 vs. 31.3, *p* < 0.001). **Conclusions:** The study provides robust evidence that a personalized rehabilitation intervention significantly improves survival, functional independence, and coping strategies in cancer patients with spinal bone metastasis.

## 1. Introduction

Cancer remains a significant global health issue in the 21st century, accounting for 16.8% of all deaths and 22.8% of noncommunicable disease (NCD) deaths globally. Among individuals aged 30–69 years, cancer is responsible for 30.3% of premature NCD deaths and ranks among the top three causes of death in 177 of 183 countries [1,2]. Beyond its immediate mortality impact, cancer survivors face long-term health consequences, including neuropathy, cardiotoxicity, and chronic conditions that compromise their quality of life and functional independence [3].

Psychological well-being, encompassing hedonic and eudaimonic constructs, has emerged as a critical determinant of health outcomes. Evidence suggests that positive psychological states, such as optimism, life satisfaction, and a sense of purpose, are not only markers of better psychosocial functioning but also uniquely contribute to physical health and reduced mortality risk [4,5,6,7,8]. A 2017 meta-analysis of 76 prospective studies demonstrated that higher levels of these attributes are consistently associated with improved survival outcomes [9].

Exercise interventions have proven effective in mitigating cancer-related fatigue (CRF) and enhancing the quality of life (QoL) of cancer patients. Aerobic exercise programs lasting less than 12 weeks, performed three times weekly, appear particularly beneficial. However, although female patients may derive greater benefits from these interventions, the reasons for this disparity remain poorly understood [9]. Furthermore, despite its well-documented benefits, exercise remains one of the least prioritized behaviors among cancer patients following diagnosis and treatment [10,11].

Furthermore, coping strategies play a pivotal role in cancer survivorship [12]. Emotion-focused strategies, which are more commonly employed by women with breast cancer, have been associated with poorer quality of life and greater pain interference, while problem-focused strategies show no such correlation [12]. Understanding these mechanisms and tailoring interventions is essential for improving patient outcomes.

Rehabilitation interventions tailored to patients with cancer have shown promise in improving functional independence, reducing disease-related complications, and enhancing coping mechanisms. These interventions are particularly critical for patients with advanced or metastatic diseases, in which the physical and psychosocial burdens are more pronounced. Despite this potential, the long-term effects of such interventions on survival, functional independence, and coping strategies remain unclear.

This study evaluated the impact of a novel integrated rehabilitation intervention designed specifically for cancer patients. By examining its effects on clinical outcomes such as survival, motor function, and pain management, as well as psychosocial outcomes, including coping strategies and quality of life, the study aimed to provide evidence for developing more effective rehabilitation protocols. The ultimate goal was to enhance patient care and improve survivorship experiences, particularly for those with advanced or metastatic diseases.

## 2. Materials and Methods

### 2.1. Study Design and Objectives

Data were collected over three years (2022–2024) at Spitalul Clinic Județean de Urgență Oradea. Eligible patients with vertebral metastasis were randomly assigned using stratified randomization to ensure balanced distribution of key prognostic factors (such as age, tumor type, or baseline functionality) between groups. Patients were then allocated to two treatment arms: one group was managed by a physician providing standard care (control group, no kinetotherapy), and the other by a physician administering personalized kinetotherapy (experimental group).

**Figure 1:**
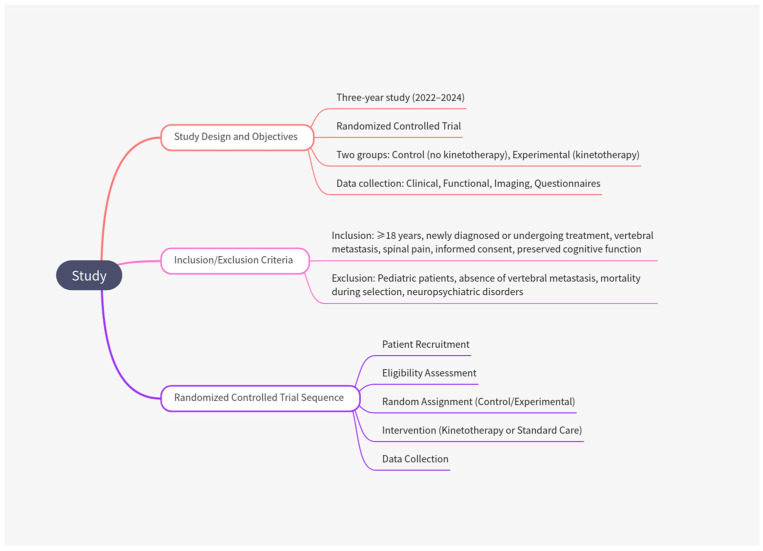
Flow diagram of study inclusion and selection.

Comprehensive clinical, functional, and imaging assessments were conducted to evaluate sources of pain, functional status, and coping mechanisms. Additionally, validated questionnaires were used to assess pain perception, quality of life, and coping strategies, ensuring consistency in data collection across both groups. Standardized protocols were rigorously implemented to minimize bias and enhance the reliability of the study findings. A flow diagram detailing study inclusion and participant selection is presented in Figure 1.

### 2.2. Outcome Measures

The outcome measures in this study were designed to capture both the clinical and psychosocial dimensions of patient health. Clinical outcomes were assessed through survival analysis, functional independence evaluations, and documentation of complications. Survival was evaluated using Kaplan–Meier survival curves and a Cox proportional hazards regression model adjusted for confounders such as age, marital status, and tumor type. Functional independence was measured using the Barthel Index and Frankel Scale, which provided both quantitative scores and categorical classifications of neurological function. Complications, including fractures, osteoporosis, anemia, and vomiting, were recorded based on clinical examinations and imaging studies. Psychosocial outcomes were measured using standardized questionnaires that evaluated pain perception, quality of life, and coping strategies. These instruments were chosen to ensure a comprehensive evaluation of both the physical and emotional aspects of patient well-being.

### 2.3. Inclusion and Exclusion Criteria

The inclusion criterion was a minimum age of 18 years, with eligible participants being newly diagnosed oncological patients or those undergoing oncological treatment. The participants were required to present with vertebral metastasis and spinal pain (rachialgia), provide informed consent, and demonstrate preserved cognitive function.

The exclusion criteria included pediatric patients, absence of vertebral metastasis, mortality during the selection period, and neuropsychiatric disorders that impaired judgment. Cognitive function was assessed through a detailed review of each patient’s psychiatric history and current medication usage. Patients with a documented history of neuropsychiatric disorders or those receiving psychiatric medications were excluded, ensuring that only patients with preserved cognitive function participated in the study.

### 2.4. Ethical Considerations

All patients provided informed consent in compliance with the Declaration of Helsinki. The study received ethical approval under the reference numbers 42571/16 December 2022 and 42371/15 December 2022.

### 2.5. Statistical Analysis

Statistical analysis for this study was performed in several stages to evaluate the impact of the intervention on survival, functional independence, and coping mechanisms among oncological patients with vertebral metastasis. Data preparation involved verifying the accuracy and completeness of the collected information, with any missing data addressed through appropriate methods such as exclusion or imputation based on clinical relevance and statistical assumptions. Continuous variables, such as age and BMI, were summarized using means, standard deviations, medians, and ranges, whereas categorical variables, including sex, environment, and education level, were reported as frequencies and percentages.

The normality of continuous variables was assessed using the Shapiro–Wilk test. For normally distributed variables, independent *t*-tests were used to compare means between the experimental and control groups, while non-parametric alternatives, such as the Mann–Whitney U test, were applied for skewed data. Categorical data were compared using chi-square tests for independence, and Fisher’s exact test was employed when cell counts were <five to ensure accuracy.

To assess the impact of the intervention on survival, Kaplan–Meier survival curves were constructed for both groups, and the log-rank test was used to compare the survival distributions. A Cox proportional hazards regression model was employed to adjust for potential confounding variables such as age, marital status, and tumor type, providing hazard ratios and 95% confidence intervals to quantify the effect of the intervention on survival outcomes.

Coping mechanisms were evaluated using psychological scales, and mean scores were compared between groups using *t*-tests or one-way ANOVA, depending on the number of groups. Multiple testing corrections, such as Bonferroni adjustment, were applied to control for the risk of Type I errors due to repeated comparisons. Factor analysis was performed for scale validation where applicable.

Finally, sensitivity analyses were conducted to examine the robustness of the findings under various assumptions regarding missing data and outliers. All statistical tests were two-tailed, with a significance threshold of *p* < 0.05. Statistical analyses were performed using R, version 4.4.2, developed by the R Core Team and sourced from the R Foundation for Statistical Computing in Vienna, Austria. The analysis included the use of the stats package for normality testing (Shapiro–Wilk test), *t*-tests, and ANOVA; the psych package for descriptive statistics and factor analysis; the coin package for Mann–Whitney U tests; and the Exact 2 × 2 package for Fisher’s exact tests. Survival analysis was performed using the survival package for Kaplan–Meier curves and Cox regression, along with the Survminer package for visualizing survival curves. Multiple testing corrections were applied using either the Multcomp package or the *p* adjust function. Missing data were addressed using imputation methods available in the mice or missForest packages.

## 3. Results

### 3.1. Baseline Characteristics

**Figure 2:**
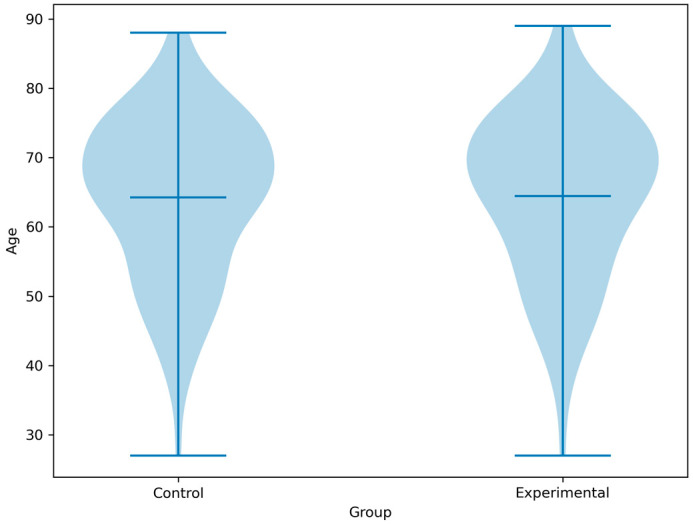
Age distribution of the control and experimental groups.

The mean age of the participants in the experimental group was 64.5 years (SD = 11.9) and 64.2 years (SD = 11.8) in the control group. The age range was similar across both groups (27–89 years) (Figure 2).

**Figure 3:**
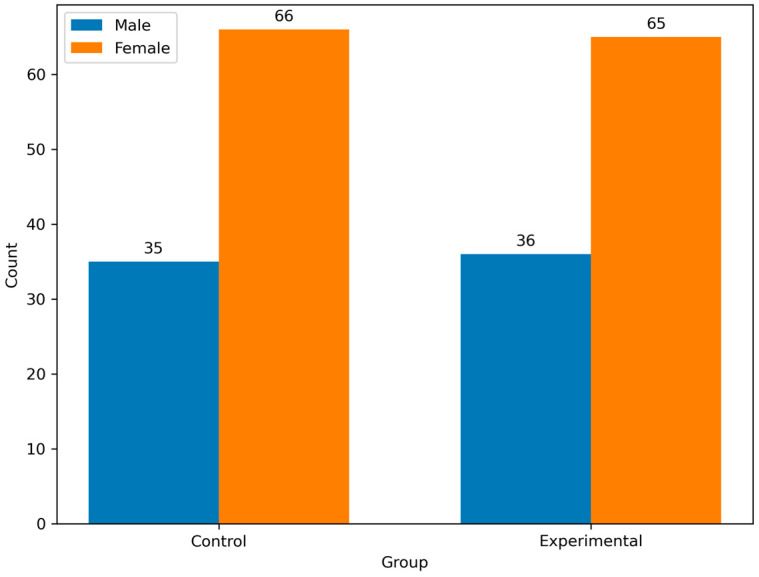
Distribution of gender by groups.

Both groups had a similar sex distribution, with females constituting 64.4% of the experimental group and 65.3% of the control group. Moreover, the proportion of men was comparable (35.6% vs. 34.7%). There were no significant differences between genders (*p* = 0.8832) (Figure 3).

The participants’ rural and urban distributions were balanced, with 40.6% and 39.6% from rural areas in the experimental and control groups, respectively, and 59.4% and 60.4%, respectively, from urban areas (*p* = 0.8862). Education levels were comparable across the groups, with slight variations in proportions across categories (e.g., gymnasium, high school, college) (*p* = 0.8652). A significant difference was noted in marital status distribution (*p* = 0.0102), with the experimental group having a higher proportion of married individuals (63.4% vs. 48.5%) and fewer unmarried (1.0% vs. 5.9%) or concubine participants (2.0% vs. 9.9%) than the control group. The mean BMI was lower in the experimental group (21.1 kg/m^2^, SD = 4.3) compared to the control group (22.7 kg/m^2^, SD = 5.3) (*p* = 0.021), with a wider range observed in the experimental group (2.5–38.9) compared to the control group (17.3–38.9). Smoking habits were evenly distributed between the two groups, with 50.5% in the experimental group and 49.5% in the control group identified as smokers (*p* = 0.8882) (Table 1).

### 3.2. Clinical Characteristics and Outcomes

#### 3.2.1. Physical Activity and Tumor Characteristics

Minimal physical activity was more frequently reported in the control group (51.5%) than in the experimental group (36.6%). Moderate activity levels were similar between the two groups, with 53.5% in the experimental group and 47.5% in the control group. Intensive physical activity was higher in the experimental group (8.9%) than in the control group (1.0%; *p* = 0.0162). Metastatic tumors were more prevalent in the experimental group (94.9%) than in the control group (67.7%), whereas primary tumors were more common in the control group (30.3%) than in the experimental group (3.0%) (*p* < 0.0012) (Table 2).

#### 3.2.2. Pain and Sphincter Disorders

The presence of pain was high in both groups but was slightly more prevalent in the experimental group (96.0%) than in the control group (86.0%). The absence of pain was more common in the control group (14.0%) than in the experimental group (4.0%; *p* = 0.0132). Constipation was observed more frequently in the control group (27.7%) compared to the experimental group (20.8%), *p* = 0.2512, though this difference was not significant. Urinary retention followed a similar trend, with a slightly higher prevalence in the control group (24.8%) than in the experimental group (15.8%; *p* = 0.2322). The incontinence rates were comparable between the two groups (experimental: 22.0%, control: 26.7%) and were not significantly different (*p* = 0.4352) (Table 3).

#### 3.2.3. Complications and Survival

Fractures were significantly more frequent in the control group (55.4%) than in the experimental group (20.0%; *p* < 0.0012). Furthermore, osteoporosis was more prevalent in the control group (37.0%) than in the experimental group (17.0%; *p* = 0.0012). Anemia showed a similar trend, with a higher prevalence in the control group (57.4%) than in the experimental group (22.2%) (*p* < 0.0012). Vomiting was much more frequent in the control group (54.5%) than in the experimental group (6.1%; *p* < 0.0012). The mean survival time was significantly longer in the experimental group (3.5 years) than in the control group (2.8 years; *p* < 0.0011). This suggests that the experimental group may have experienced better survival outcomes, potentially influenced by the treatment or baseline characteristics (Table 4).

### 3.3. Logistic Regression Analysis

The logistic regression model investigates the relationship between various neurological and sphincter-related conditions and the likelihood of a patient being assigned to the control group relative to the experimental group. The model’s intercept, with an estimate of 0.691 (SE = 0.568, z = 1.217, *p* = 0.224), is not statistically significant, indicating that, in the absence of the predictors, the baseline odds of group membership do not differ significantly.

Localized pain, defined as the presence versus absence of pain in a specific area, shows an estimate of −0.438 (SE = 0.455, z = −0.964, *p* = 0.335) and an odds ratio of 0.645, suggesting a non-significant trend toward lower odds of being in the control group among those with localized pain. Radiating pain, which may indicate a more diffuse pain pattern, has an estimate of −0.702 (SE = 0.370, z = −1.898, *p* = 0.058) with an odds ratio of 0.496. Although this result approaches statistical significance, it does not meet the conventional threshold (*p* < 0.05).

Among the motor deficits, monoplegia is significantly associated with group membership. Patients with monoplegia have a significantly lower likelihood of being in the control group, as indicated by an estimate of −1.368 (SE = 0.470, z = −2.911, *p* = 0.004) and an odds ratio of 0.255. Hemiparesis demonstrates an even stronger association, with an estimate of −1.927 (SE = 0.568, z = −3.391, *p* < 0.001) and an odds ratio of 0.146, signifying that the presence of hemiparesis markedly decreases the odds of being in the control group. Tetraparesis exhibits a significant borderline effect (Estimate = −1.615, SE = 0.825, z = −1.956, *p* = 0.050), with an odds ratio of 0.199, suggesting a potential trend toward lower odds of being in the control group among those with tetraparesis.

Paresthesia, representing abnormal sensations, yields a non-significant estimate of 0.205 (SE = 0.335, z = 0.613, *p* = 0.540), with an odds ratio of 1.228, indicating little difference between groups. Conversely, hypoesthesia shows a statistically significant positive association (Estimate = 0.795, SE = 0.355, z = 2.238, *p* = 0.025), with an odds ratio of 2.215. This implies that patients exhibiting hypoesthesia are more than twice as likely to be in the control group compared to the experimental group. Anesthesia and hypersensitivity, with estimates of 0.359 (*p* = 0.396) and 0.609 (*p* = 0.204), respectively, do not reach statistical significance.

Regarding sphincter disorders, the conditions of constipation, urinary retention, and incontinence yield non-significant results, with odds ratios of 1.605, 1.722, and 1.480, respectively. Finally, motor deficit due to monoparesis presents a non-significant estimate of −0.442 (*p* = 0.225) and an odds ratio of 0.643, suggesting that this factor does not significantly differentiate between the groups.

The analysis reveals that among the variables examined, monoplegia and hemiparesis are significantly associated with a reduced likelihood of being in the control group, while hypoesthesia is significantly associated with an increased likelihood. The remaining predictors, including localized and radiating pain, tetraparesis, paresthesia, anesthesia, hypersensitivity, sphincter disorders, and motor deficit due to monoparesis, do not demonstrate statistically significant associations with group membership. These findings indicate that specific motor impairments, particularly monoplegia and hemiparesis, are key differentiators between the two groups, whereas the presence of hypoesthesia appears to favor assignment to the control group. The overall results should be interpreted within the context of the study’s design and sample sizes, which comprised 101 patients in the control group and 101 patients in the experimental group (Table 5).

**Figure 4:**
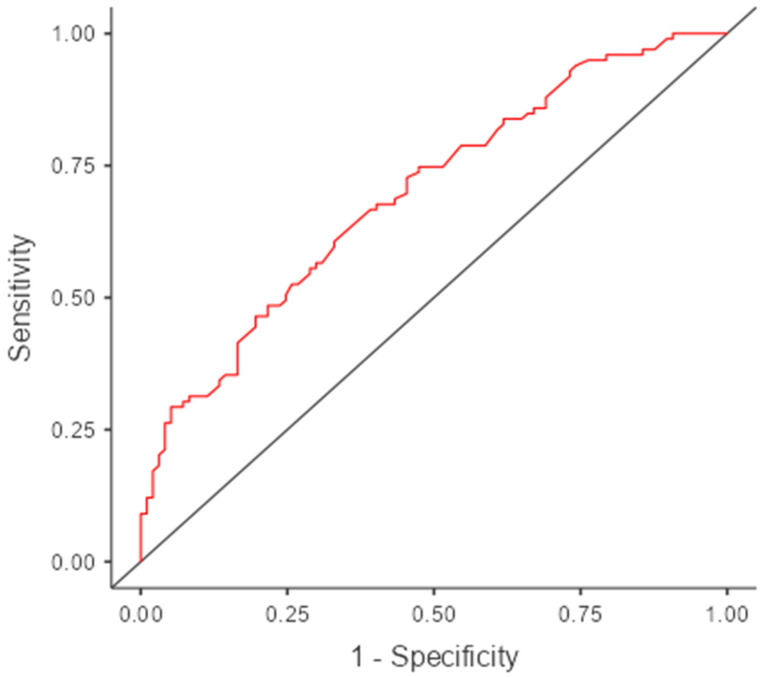
Classification Performance and ROC Analysis of the Predictive Model.

The classification results indicate that among patients observed as experimental, 55 out of 97 (56.7%) were correctly predicted, while for those observed as control, 67 out of 99 (67.7%) were correctly classified. The overall accuracy of the model is 0.622, which suggests that approximately 62.2% of the cases were correctly classified using the cut-off of 0.5. The specificity of 0.567 reflects a moderate ability of the model to correctly identify patients in the control group, whereas the sensitivity of 0.677 indicates a slightly higher capacity to correctly classify patients in the experimental group. The area under the ROC curve (AUC) is 0.696, demonstrating acceptable discriminative power of the model (Figure 4). Together, these findings suggest that while the model performs moderately well in distinguishing between the two groups, there is room for improvement, particularly in enhancing specificity and overall classification accuracy (Table 6).

### 3.4. Functional and Neurological Assessments

The Barthel Total Score was significantly higher in the experimental group (mean: 60.7) than in the control group (mean: 40.8) (*p* = 0.0021). The distribution across the Frankel Scale categories was identical between the groups, with no significant differences (*p* = 1.0002). Most patients were classified into the highest functional category (E: 41.6%), followed by category D (28.7%), indicating a generally high level of neurological function (Table 7).

Table 8 presents a detailed analysis of motor deficits categorized into domains such as monoparesis, monoplegia, hemiparesis, tetraparesis, and paresthesia, comparing their prevalence between the experimental and control groups. Each domain was analyzed individually, with statistical significance levels noted where applicable.

The prevalence of monoparesis was nearly identical between the experimental (49.5%) and control (51.5%) groups, with no significant difference (*p* = 0.7782). Monoplegia was slightly more common in the control group (21.8%) than in the experimental group (11.9%); however, this difference was not statistically significant (*p* = 0.1092). A severe form of monoplegia (category 2) was observed exclusively in the control group, although it was rare, affecting only one individual (1.0%). Hemiparesis was significantly more prevalent in the experimental group (20.0%) than in the control group (8.9%) (*p* = 0.0252). Missing data were minimal, with only one instance, ensuring a robust analysis.

Tetraparesis was similarly distributed between the experimental (5.0%) and control (4.0%) groups, with no significant difference observed (*p* = 0.7332). Paresthesia was slightly more prevalent in the control group (56.4%) than in the experimental group (53.5%); however, the difference was not statistically significant (*p* = 0.6712).

### 3.5. Coping Strategies by Gender

Table 9 compares coping strategies between the experimental and control groups, focusing on females, males, and the total mean scores.

In the domain of Positive Interpretation and Growth, the experimental group recorded a total mean of 9.7 (2.1), with no significant gender differences (*p* = 0.7171). The control group showed a similar trend, with a total mean of 9.3 (1.6), with no significant difference (*p* = 0.7981).

For Mental Disengagement, the experimental group scored 7.7 (2.3) for females and 7.8 (1.8) for males (*p* = 0.9791). The control group recorded slightly higher scores, with a mean of 8.4 (1.9), although this difference was not statistically significant (*p* = 0.7621).

In the domain of Focus on Emotional Expression, the experimental group showed a significant gender difference, with females scoring higher (12.1 ± 2.0) than males (9.7 ± 1.7), resulting in a *p*-value of less than 0.001. Similarly, in the control group, females scored 12.5 (1.5) compared to males at 11.1 (1.8), also with a *p*-value of less than 0.001.

The experimental and control groups showed comparable scores for the use of Instrumental Social Support. In the experimental group, the total mean was 13.1 (2.1), with no significant gender differences (*p* = 0.1981). The control group recorded a mean of 13.1 (1.7), with no significant differences (*p* = 0.7011).

For the Religious Approach, females in the experimental group scored significantly higher (14.8 ± 2.0) than males (13.7 ± 1.7), with a *p*-value of 0.0081. In the control group, a similar gender difference was observed, with females scoring 14.4 (1.8) compared to males at 13.6 (1.5), resulting in a *p*-value of 0.0311. These findings highlight the preference for religious coping strategies among females in both groups.

In the Social Support Coping domain, the experimental group recorded a total mean of 34.9 (4.8), with significant gender differences (*p* < 0.0011). The control group, with a mean of 35.5 (3.4), also showed a significant difference (*p* = 0.0011). Social support as a coping strategy was significantly different between genders and across the groups.

The results showed that significant differences were present in specific domains, including Focus on Emotional Expression, Religious Approach, and Social Support Coping, with females scoring consistently higher across these areas. Domains such as Positive Interpretation and Growth, Mental Disengagement, and Denial exhibited similar scores between the groups, reflecting non-significant differences. The experimental group demonstrated slightly greater reliance on certain coping strategies, which may reflect the influence of the intervention. These findings emphasize the importance of tailoring interventions to sex-specific coping patterns, particularly in strategies related to emotional and social support.

## 4. Discussion

The aim of this study was to evaluate the impact of a tailored rehabilitation intervention on clinical and psychosocial outcomes in cancer patients with vertebral metastasis. Patients were divided into a control group, which received standard care without kinetotherapy, and an experimental group, which underwent personalized kinetotherapy interventions. The results demonstrated that the intervention significantly improved functional independence, survival, and coping mechanisms, despite the experimental group having a higher prevalence of metastatic disease and hemiparesis. These findings underscore the effectiveness of personalized rehabilitation interventions in addressing the complex needs of cancer patients, particularly those with advanced diseases.

Psychosocial interventions have shown varying degrees of effectiveness in cancer care, with some studies reporting significant benefits, while others note limited or no effects. This study’s findings align with evidence suggesting that interventions targeting mental health and quality of life can yield meaningful improvements. For instance, emotional expression, muscle relaxation training, and self-efficacy enhancement have been shown to positively influence mental health outcomes in colorectal cancer patients [13]. Similarly, cognitive behavioral therapy and family therapy have proven effective in pediatric oncology, reducing anxiety and depression while enhancing quality of life [14]. However, inconsistent findings in previous studies, such as the lack of significant changes in quality of life or psychological scores in breast cancer patients following psychosocial interventions, underscore the variability of outcomes and the need for tailored approaches [15].

This study adds to the existing body of literature by evaluating the personalized kinetotherapy intervention designed specifically for cancer patients with advanced or metastatic disease. The significant improvements observed in functional independence and survival suggest that this intervention may address limitations in previous studies, particularly by integrating physical, psychological, and social components. Furthermore, the results reinforce prior findings that adaptive coping strategies, such as emotional and social support, are associated with better outcomes, while disengaged coping strategies predict poorer quality of life and greater psychological distress [16,17].

The experimental group demonstrated better outcomes despite a higher prevalence of metastatic disease and hemiparesis, which are typically associated with worse prognoses. These findings may reflect the intervention’s comprehensive nature, targeting both physical and psychosocial aspects of patient care. One possible explanation is that the intervention facilitated better adaptation to disease-related challenges, enabling patients to maintain or improve their functional independence and overall well-being. This hypothesis aligns with evidence that structured physical activity and psychosocial support can buffer the negative effects of advanced disease [18].

The differences in coping mechanisms further illustrate the intervention’s impact. The experimental group exhibited lower reliance on avoidant coping strategies, such as denial and behavioral disengagement, which are known to exacerbate emotional distress. In contrast, the control group’s higher scores in these domains may have contributed to their poorer outcomes. These findings are consistent with studies linking disengaged coping to worse quality of life and increased anxiety and depression in cancer patients [16,17]. Furthermore, religious/spiritual coping was minimally assessed in this study, which could have limited the detection of spiritual pain or maladaptive religious coping mechanisms that have been linked to greater emotional distress in other research [19].

This study highlights the importance of integrating tailored rehabilitation interventions into cancer care. The findings suggest that comprehensive approaches addressing both physical and psychosocial dimensions can significantly enhance patient outcomes. Practical applications include designing multidisciplinary programs that incorporate elements of physical activity, emotional support, and coping strategy training. For example, interventions could focus on reducing reliance on avoidant coping mechanisms while promoting adaptive strategies, such as acceptance, emotional expression, and social support [20,21,22].

Additionally, the significant improvements in survival and functional independence observed in this study underscore the need to expand access to such interventions, particularly for patients with advanced diseases. By addressing the specific needs of this population, healthcare providers can improve not only clinical outcomes but also the overall quality of life for cancer patients.

## 5. Limitations and Future Directions

Although the results of this study are promising, several limitations must be acknowledged. The relatively small sample size may limit the generalizability of our findings, particularly for subgroups with less common diagnoses or characteristics. Additionally, the lack of long-term follow-up data prevents a comprehensive assessment of the sustained effects of the intervention on survival and QoL. Future research should prioritize larger multicenter trials to validate these findings and explore the mechanisms underlying the observed benefits [16].

Further studies should investigate the potential of integrating social and treatment center variables into rehabilitation interventions. While this study focused primarily on individual-level outcomes, addressing systemic factors could provide additional insights into optimizing the care for cancer patients. Finally, tailoring interventions to specific cancer types and patient populations remains an important avenue for future research, as different groups may benefit from distinct approaches [15].

## 6. Conclusions

This study provides valuable evidence for the efficacy of a novel rehabilitation intervention to improve functional independence, survival, and coping mechanisms in patients with cancer. Addressing gaps in the existing literature underscores the importance of comprehensive, tailored approaches to cancer care. These findings have important implications for future treatment and rehabilitation strategies, highlighting the need for continued innovation to address the complex needs of patients with cancer. Further research is needed to fully understand the long-term benefits and mechanisms of such interventions and ensure their effective integration into routine clinical practice.

## Figures and Tables

**Table 1:** Baseline characteristics of the control and experimental group.

	Experimental (*N* = 101)	Control (*N* = 101)	Total (*N* = 202)	*p* Value
Age				0.892 ^1^
Mean (SD)	64.5 (11.9)	64.2 (11.8)	64.3 (11.8)	
Range	27.0–89.0	27.0–88.0	27.0–89.0	
Sex (M/F)				0.883 ^2^
Female	65.0 (64.4%)	66.0 (65.3%)	131.0 (64.9%)	
Male	36.0 (35.6%)	35.0 (34.7%)	71.0 (35.1%)	
Environment (U/R)				0.886 ^2^
Rural	41.0 (40.6%)	40.0 (39.6%)	81.0 (40.1%)	
Urban	60.0 (59.4%)	61.0 (60.4%)	121.0 (59.9%)	
Education				0.865 ^2^
Gymnasium	13.0 (12.9%)	14.0 (13.9%)	27.0 (13.4%)	
Professional	28.0 (27.7%)	26.0 (25.7%)	54.0 (26.7%)	
Highschool	31.0 (30.7%)	30.0 (29.7%)	61.0 (30.2%)	
Collage	28.0 (27.7%)	31.0 (30.7%)	59.0 (29.2%)	
Marital Status				0.010 ^2^
Unmarried	1.0 (1.0%)	6.0 (5.9%)	7.0 (3.5%)	
Married	64.0 (63.4%)	49.0 (48.5%)	113.0 (55.9%)	
Divorced	11.0 (10.9%)	18.0 (17.8%)	29.0 (14.4%)	
Concubine	2.0 (2.0%)	10.0 (9.9%)	12.0 (5.9%)	
Widow	23.0 (22.8%)	18.0 (17.8%	41.0 (20.3%)	
BMI kg/m^2^				0.021 ^1^
Mean (SD)	21.1 (4.3)	22.7 (5.3)	21.9 (4.9)	
Range	2.5–38.9	17.3–38.9	2.5–38.9	
Smoking status				0.888 ^2^
Non-smoker	51.0 (50.5%)	50.0 (49.5%)	101.0 (50.0%)	
Smoker	50.0 (49.5%)	51.0 (50.5%)	101.0 (50.0%)	

^1^ Linear Model ANOVA, ^2^ Pearson Chi-Square Test.

**Table 2:** Physical Activity and Tumor Characteristics.

Variable	Experimental (*N* = 101)	Control (*N* = 101)	Total (*N* = 202)	*p*-Value
Previous Physical Activity				0.0162
Minimal	37 (36.6%)	52 (51.5%)	89 (44.1%)	
Moderate	54 (53.5%)	48 (47.5%)	102 (50.5%)	
Intensive	9 (8.9%)	1 (1.0%)	10 (5.0%)	
Diagnosis (Primary/Metastatic)				<0.0012
Primary Tumor	3 (3.0%)	30 (30.3%)	33 (16.7%)	
Metastatic Tumor	94 (94.9%)	67 (67.7%)	161 (81.3%)	
Undetermined Tumor	0 (0.0%)	1 (1.0%)	1 (0.5%)	

**Table 3:** Pain and Sphincter Disorders.

Variable	Experimental (*N* = 101)	Control (*N* = 101)	Total (*N* = 202)	*p*-Value
Pain				0.0132
No Pain	4 (4.0%)	14 (14.0%)	18 (9.0%)	
Pain Present	96 (96.0%)	86 (86.0%)	182 (91.0%)	
Sphincter Disorders				
Constipation	21 (20.8%)	28 (27.7%)	49 (24.3%)	0.2512
Urinary Retention	16 (15.8%)	25 (24.8%)	41 (20.3%)	0.2322
Incontinence	22 (22.0%)	27 (26.7%)	49 (24.4%)	0.4352

**Table 4:** Complications and Survival.

Variable	Experimental (*N* = 101)	Control (*N* = 101)	Total (*N* = 202)	*p*-Value
Complications				
Fractures	20 (20.0%)	56 (55.4%)	76 (37.8%)	<0.0012
Osteoporosis	17 (17.0%)	37 (37.0%)	54 (27.0%)	0.0012
Anemia	22 (22.2%)	58 (57.4%)	80 (40.0%)	<0.0012
Vomiting	6 (6.1%)	55 (54.5%)	61 (30.5%)	<0.0012
Survival Time (Years)				<0.0011
Mean (SD)	3.5 (1.3)	2.8 (1.1)	3.1 (1.3)	
Range	0.0–7.0	0.0–5.0	0.0–7.0	

**Table 5:** Logistic Regression Model Coefficients—Association with Group Membership (Control vs. Experimental).

Predictor	Estimate	SE	Z	*p*	Odds Ratio	95% CI Lower	95% CI Upper
Intercept	0.691	0.568	1.217	0.224	1.997	0.6558	6.079
Localized pain (Yes vs. No)	−0.438	0.455	−0.964	0.335	0.645	0.2647	1.572
Radiating pain (Yes vs. No)	−0.702	0.37	−1.898	0.058	0.496	0.2403	1.023
Monoplegia (Yes vs. No)	−1.368	0.47	−2.911	0.004	0.255	0.1014	0.64
Hemiparesis (Yes vs. No)	−1.927	0.568	−3.391	<0.001	0.146	0.0478	0.443
Tetraparesis (Yes vs. No)	−1.615	0.825	−1.956	0.05	0.199	0.0394	1.003
Paresthesia (Yes vs. No)	0.205	0.335	0.613	0.54	1.228	0.6366	2.369
Hypoesthesia (Yes vs. No)	0.795	0.355	2.238	0.025	2.215	1.1039	4.445
Anesthesia (Yes vs. No)	0.359	0.424	0.848	0.396	1.432	0.6244	3.286
Hypersensitivity (Yes vs. No)	0.609	0.48	1.269	0.204	1.838	0.718	4.704
Sphincter Disorders—Constipation (Yes vs. No)	0.473	0.397	1.192	0.233	1.605	0.7373	3.495
Sphincter Disorders—Urinary Retention (Yes vs. No)	0.543	0.415	1.308	0.191	1.722	0.7627	3.887
Sphincter Disorders—Incontinence (Yes vs. No)	0.392	0.425	0.922	0.357	1.48	0.643	3.407
Motor Deficit—Monoparesis (Yes vs. No)	−0.442	0.364	−1.214	0.225	0.643	0.3147	1.312

**Table 6:** Confusion Matrix and Predictive Measures (Cut-off = 0.5).

Category	Predicted Experimental	Predicted Control	% Correct
Observed Experimental (*n* = 97)	55	42	56.70%
Observed Control (*n* = 99)	32	67	67.70%
Predictive Measures			
Accuracy	0.622		
Specificity	0.567		
Sensitivity	0.677		
AUC	0.696		

**Table 7:** Functional Scores.

Variable	Experimental (*N* = 101)	Control (*N* = 101)	Total (*N* = 202)	*p*-Value
Barthel Total Score				0.0021
Mean (SD)	60.7 (61.4)	40.8 (21.9)	50.8 (47.1)	
Range	0.0–585.0	0.0–70.0	0.0–585.0	
Frankel Scale				1.0002
A	3 (3.0%)	3 (3.0%)	6 (3.0%)	
B	12 (11.9%)	12 (11.9%)	24 (11.9%)	
C	15 (14.9%)	15 (14.9%)	30 (14.9%)	
D	29 (28.7%)	29 (28.7%)	58 (28.7%)	
E	42 (41.6%)	42 (41.6%)	84 (41.6%)	

**Table 8:** Comparison of Motor Deficit Prevalence Between Experimental and Control Groups.

Monoparesis				0.778 ^2^
No	50.0 (49.5%)	52.0 (51.5%)	102.0 (50.5%)	
Yes	51.0 (50.5%)	49.0 (48.5%)	100.0 (49.5%)	
Monoplegia				0.109 ^2^
No	79.0 (78.2%)	88.0 (87.1%)	167.0 (82.7%)	
Yes	22.0 (21.8%)	12.0 (11.9%)	34.0 (16.8%)	
N-Miss	0.0 (0.0%)	1.0 (1.0%)	1.0 (0.5%)	
Hemiparesis				0.025 ^2^
N-Miss	1.0	0.0	1.0	
No	80.0 (80.0%)	92.0 (91.1%)	172.0 (85.6%)	
Yes	20.0 (20.0%)	9.0 (8.9%)	29.0 (14.4%)	
Tetraparesis				0.733 ^2^
No	96.0 (95.0%)	97.0 (96.0%)	193.0 (95.5%)	
Yes	5.0 (5.0%)	4.0 (4.0%)	9.0 (4.5%)	
Paresthesia				0.671 ^2^
No	47.0 (46.5%)	44.0 (43.6%)	91.0 (45.0%)	
Yes	54.0 (53.5%)	57.0 (56.4%)	111.0 (55.0%)	

^2^ Pearson Chi-Square Test.

**Table 9:** Comparative Analysis of Coping Strategies Between Experimental and Control Groups by Gender.

Domain	Experimental Group	Experimental Group	Experimental Group	Experimental Group	Control Group	Control Group Male	Control Group Total	Control Group
	Female	Male	Total Mean	*p*-Value	Female			*p*-Value
Positive Interpretation and Growth	9.6 (2.2)	9.8 (2.0)	9.7 (2.1)	0.7171	9.2 (1.7)	9.3 (1.5)	9.3 (1.6)	0.7981
Mental Disengagement	7.7 (2.3)	7.8 (1.8)	7.7 (2.1)	0.9791	8.4 (1.9)	8.3 (2.0)	8.4 (1.9)	0.7621
Focus on Emotional Expression	12.1 (2.0)	9.7 (1.7)	11.2 (2.2)	<0.0011	12.5 (1.5)	11.1 (1.8)	12.0 (1.8)	<0.0011
Use of Instrumental Social Support	13.3 (2.1)	12.8 (2.1)	13.1 (2.1)	0.1981	13.1 (1.7)	13.0 (1.5)	13.1 (1.7)	0.7011
Active Approach	12.4 (2.2)	12.1 (1.8)	12.3 (2.1)	0.5931	12.6 (1.7)	12.5 (1.8)	12.6 (1.7)	0.6191
Denial	11.1 (1.9)	11.1 (2.1)	11.1 (1.9)	0.9881	11.4 (1.6)	11.2 (1.7)	11.3 (1.6)	0.5721
Religious Approach	14.8 (2.0)	13.7 (1.7)	14.4 (2.0)	0.0081	14.4 (1.8)	13.6 (1.5)	14.1 (1.7)	0.0311
Humor	5.8 (2.1)	6.8 (3.6)	6.2 (2.8)	0.0841	6.1 (2.1)	6.5 (2.3)	6.2 (2.2)	0.3241
Behavioral Disengagement	10.2 (2.2)	10.1 (1.9)	10.2 (2.1)	0.9451	11.5 (1.9)	11.7 (1.9)	11.6 (1.9)	0.5621
Restraint	10.3 (1.7)	9.9 (1.8)	10.1 (1.8)	0.2761	10.6 (1.6)	10.6 (1.5)	10.6 (1.5)	0.9821
Use of Emotional Social Support	10.9 (2.4)	9.8 (1.9)	10.5 (2.3)	0.0221	10.6 (1.8)	9.9 (1.6)	10.4 (1.8)	0.0371
Substance Use	5.6 (2.9)	6.2 (2.5)	5.8 (2.8)	0.2631	5.5 (2.3)	6.4 (2.4)	5.8 (2.3)	0.0501
Acceptance	10.2 (3.2)	10.1 (2.5)	10.2 (3.0)	0.8831	10.4 (2.0)	10.3 (1.8)	10.3 (1.9)	0.8161
Suppression of Competing Activities	11.3 (2.3)	10.8 (2.2)	11.1 (2.2)	0.2341	11.2 (1.8)	10.5 (1.4)	11.0 (1.7)	0.0321
Planning	11.1 (2.5)	11.2 (2.0)	11.2 (2.3)	0.8631	10.5 (2.0)	10.7 (1.9)	10.6 (2.0)	0.6801
Problem-Focused Coping	34.8 (5.5)	34.1 (4.4)	34.6 (5.1)	0.5121	34.4 (4.2)	33.6 (3.3)	34.1 (3.9)	0.3501
Emotion-Focused Coping	30.2 (4.3)	29.8 (3.9)	30.0 (4.1)	0.6981	30.2 (3.4)	30.2 (2.7)	30.2 (3.2)	0.9981
Social Support Coping	36.3 (4.9)	32.3 (3.7)	34.9 (4.8)	<0.0011	36.2 (3.4)	34.0 (2.8)	35.5 (3.4)	0.0011
Avoidant Coping	29.0 (3.9)	28.9 (3.9)	29.0 (3.9)	0.9761	31.3 (3.6)	31.2 (3.3)	31.3 (3.5)	0.9031

## Data Availability

The data supporting this study are available from the corresponding author upon reasonable request.
